# Detectability of pulmonary ossifications in fibrotic lung on ultra-high-resolution CT using 2048 matrix size and 0.25-mm slice thickness

**DOI:** 10.1038/s41598-021-94596-5

**Published:** 2021-07-23

**Authors:** Akinori Hata, Masahiro Yanagawa, Mitsuko Tsubamoto, Shuhei Doi, Yuriko Yoshida, Tomo Miyata, Noriko Kikuchi, Nobuo Kashiwagi, Noriyuki Tomiyama

**Affiliations:** 1 Department of Diagnostic and Interventional Radiology, Osaka University Graduate School of Medicine, 2-2 Yamadaoka, Suita, Osaka 565-0871 Japan; 2 Department of Radiology, Nishinomiya Municipal Hospital, 8-24 Hayashida-cho, Nishinomiya, Hyogo 663-8014 Japan; 3 Department of Future Diagnostic Radiology, Osaka University Graduate School of Medicine, 2-2 Yamadaoka, Suita, Osaka 565-0871 Japan

**Keywords:** Diseases, Respiratory tract diseases

## Abstract

To investigate the prevalence of nodular pulmonary ossifications (POs) in patients with honeycombing on ultra-high-resolution CT (UHRCT) and to compare the detectability of nodular POs between images reconstructed using the ultra-high-resolution setting (UHR-setting) and those using the conventional setting (C-setting) on UHRCT. Twenty patients with honeycombing in the lung were evaluated retrospectively. All patients underwent non-contrast-enhanced UHRCT. Images were reconstructed with UHR-setting (matrix, 2048 × 2048; slice thickness, 0.25 mm) and with C-setting (matrix size, 512 × 512; slice thickness, 0.5 mm). Two chest radiologists independently recorded the number of nodular POs (< 4 mm diameter) in each lung lobes. Each lobe was classified as one of the following five categories according to the number of POs: C0, none; C1, 1–4 POs; C2, 5–9 POs; C3, 10–49 POs; and C4, ≥ 50 POs. The maximum CT values of the POs were measured and compared between the two settings. PO categories were significantly higher with UHR-setting than with C-setting (*p* < 0.001). Maximum CT values were significantly higher with UHR-setting than with C-setting (*p* < 0.001). Nodular POs were seen in 80% or more of patients with honeycombing and more easily detected in images reconstructed with UHR-setting than in those with C-setting.

## Introduction

Calcification and ossification are important diagnostic findings in thoracic computed tomography (CT). They contribute to differentiating between malignant and benign lung nodules^1^ and to identifying invasive thymomas^2^. Several studies have shown an association between diffuse pulmonary ossification (DPO) and fibrosing interstitial lung disease (ILD)^3–7^. In addition, Egashira et al. reported a significantly higher prevalence of pulmonary ossification (PO) in patients with idiopathic pulmonary fibrosis (IPF) than in those with other fibrosing ILD^8^. According to their report, PO may have a potential to differentiate IPF and the other ILD.

In the detection of calcification and ossification, spatial resolution and slice thickness are important imaging parameters. It is well known that calcification in breast cancer can be adequately depicted on mammography but not on CT due to differences in spatial resolution between these modalities. In the clinical situation, small calcifications are commonly undetectable on thick-slice images but are visible on thin-slice images. The visibility of small calcifications is affected by the partial volume effect in thick-slice images and is poor in images of low spatial resolution.

Ultra-high-resolution CT (UHRCT) scanners with 0.25 × 0.25 mm detector elements and beam collimation of 0.25 mm × 160 rows have recently become available in clinical practice^9–14^. This system can provide images with matrix size as large as 2048 × 2048 and slice thickness of 0.25 mm, which greatly increase resolution in the x–y axes and z axis^11^. We hypothesized that UHRCT images reconstructed with a high-resolution setting would have improved detectability of calcification and ossification compared with images reconstructed with a conventional setting. If PO is more detectable on UHRCT, UHRCT may contribute to the diagnosis of IPF.

The purpose of this study was to investigate the prevalence of nodular POs in patients with honeycombing on UHRCT and to compare the detectability of nodular POs between images reconstructed using the ultra-high-resolution setting (UHR-setting) and those using the conventional setting (C-setting) on UHRCT.

## Materials and methods

### Patients

This retrospective study was approved and the written informed consent was waived by the "Observational Study Ethics Review Committee, Osaka University Hospital, Osaka University, Japan " because of the retrospective study design. All the methods were carried out in accordance with relevant guidelines and regulations. Included were 20 patients (8 women, 12 men; mean age, 69.4 ± 12.1 years) who underwent CT scanning for the evaluation of interstitial lung disease between April and June 2018 at our institution. The inclusion criteria were as follows: non-contrast enhanced CT was performed with a UHRCT scanner (Aquilion Precision™; Canon Medical Systems Corp., Otawara, Tochigi, Japan), raw CT data were available, UHRCT was positive for honeycombing, and no prior lung surgery.

The diagnosis of ILD was categorized as follows: idiopathic pulmonary fibrosis (IPF), n = 12; ILD associated with a collagen-vascular disease, n = 7 (rheumatoid arthritis, n = 2; Sjögren’s syndrome, n = 2; systemic sclerosis, n = 2; polymyositis, n = 1), and interstitial pneumonia with autoimmune features (anti–myxovirus resistance-1, n = 1). The diagnosis of IPF was based on the diagnostic criteria of the 2018 American Thoracic Society/European Respiratory Society guidelines^15^. Definite usual interstitial pneumonia (UIP) pattern is defined as reticular opacities and honeycombing with subpleural and basal predominance. A patient who showed definite UIP pattern on CT without any other known causes of ILD was diagnosed as IPF without requiring surgical lung biopsy. The diagnosis for all patients in this study was based on clinical information and CT imaging. No patient had pathological proof.

### CT acquisition and image reconstruction

All CT images used in this study were obtained with a UHRCT scanner (Aquilion Precision) using the routine clinical protocol for non-contrast CT of the chest. The protocol was as follows: gantry rotation period, 0.5 s; detector collimation, 0.25 mm; focus, 0.6 × 0.6 mm; tube voltage, 120 kVp; automatic tube current modulation (standard deviation 12.0); detector pitch, 0.806 (helical pitch, 129); 1792 channels and 0.25 mm × 160 rows; and super-high-resolution mode. Images were reconstructed using each of C-setting and UHR-setting. The C-setting protocol was as follows: matrix size, 512 × 512; slice thickness, 0.5 mm; field of view (FOV), 345 mm; reconstruction kernel, FC51; and adaptive iterative dose reduction 3D (AIDR3D), eMild. The UHR-setting C protocol was as follows: matrix size, 2048 × 2048; slice thickness, 0.25 mm; FOV, 345 mm; reconstruction kernel, FC51; and AIDR3D, eStrong. Because the UHR-setting tends to produce noisy images, we conducted a preliminary evaluation of various reconstruction kernel and AIDR3D settings and selected the strong AIDR3D setting for use as it most effectively reduced noise in these reconstructions.

### Subjective PO detection

Two chest radiologists (Reader 1, M.T. with 24 years of radiology experience; and Reader 2 A.H. with 8 years of radiology experience) independently reviewed the CT images on bone window setting (level, 500 HU; width, 2500 HU) and recorded the number of nodular POs (< 4 mm diameter)^8^. The number was recorded for each of the six lung lobes (including the lingula). The C-setting images were reviewed first, followed by the UHR-setting images after an interval of at least two weeks. Each lobe was classified according to the number of nodular POs, as follows: C0, none; C1, 1–4 POs; C2, 5–9 POs; C3, 10–49 POs; and C4, ≥ 49 POs. If the patient had at least one lobe that contained at least five bilateral ossifications (C2 or higher), the patient was further classified as diffuse pulmonary ossification (DPO)^8^.

### Maximum CT number analysis

As an additional objective evaluation, maximum CT number was compared between C-setting and UHR-setting. Lobes that were categorized differently between C-setting and UHR-setting were identified for each reader. Reader 2 reviewed the identified lobes by comparing the images for each setting side-by-side. Reader 2 also identified one PO lesion in each lobe that was detectable with one setting but not with the other setting. The investigator placed a circular region of interest (ROI) with about 5-mm diameter on the area including the PO, avoiding other PO lesions and high-density architectures. The same-sized ROI was manually placed in the almost same location for both settings. The maximum CT value in the ROI was recorded for each setting. It was assumed that the maximum CT value was not to be affected by the subtle difference in the position of the ROI.

### Statistical analysis

All statistical analyses were performed using R Version 3.4.1 software. In the subjective PO detection analysis, interobserver agreement regarding the category of each lobe was assessed by weighted kappa agreement. The difference in prevalence of DPO between C-setting and UHR-setting was assessed by McNemar’s test. In each setting, the difference in categories among the upper, middle/lingula, and lower lobes were tested by Kruskal–Wallis test with post hoc testing using the Steel–Dwass test. The difference in categories between C-setting and UHR-setting was tested by Wilcoxon signed rank test. The categories of the six lobes were summed in each patient, and the difference of the summed category between IPF and the other ILDs was tested by Mann–Whitney U test. In the analysis of maximum CT number, the difference was tested by paired t-test.

We categorized the weighted kappa coefficient (κw) as poor (0 to ≤ 0.20), fair (0.20 to ≤ 0.40), moderate (0.40 to ≤ 0.60), good (0.60 to ≤ 0.80), and excellent (0.80 to ≤ 1.00)^16^. A *p*-value of < 0.05 was considered significant.

## Results

Interobserver agreement between Readers 1 and 2 for PO category was excellent (κw = 0.80) with C-setting and moderate (κw = 0.56) with UHR-setting. Table 1 lists the number of each of the PO categories, for each lobe.

**Table 1:** Results of subjective PO detection categories for each lobe.

	Reader1		Reader2	
	C-setting	UHR-setting	C-setting	UHR-setting
Total	85/25/5/2/3	22/40/26/27/5	75/34/5/3/3	57/39/9/11/4
Right upper lobe	15/3/1/1/0	1/12/3/4/0	14/4/1/1/0	9/9/0/2/0
Right middle lobe	19/1/0/0/0	8/8/3/1/0	16/4/0/0/0	12/6/1/1/0
Right lower lobe	9/6/3/0/2	1/3/4/10/2	7/8/3/0/2	4/9/2/3/2
Left upper lobe	17/3/0/0/0	4/8/6/2/0	14/6/0/0/0	13/4/1/2/0
Left lingula lobe	18/2/0/0/0	7/8/5/0/0	19/1/0/0/0	15/4/0/1/0
Left lower lobe	7/10/1/1/1	1/1/5/10/3	5/11/1/2/1	4/7/5/2/2

Data show frequency of each category (C0/C1/C2/C3/C4): C0, no PO; C1, 1–4 POs; C2, 5–9 POs; C3, 10–49 POs; and C4, more than 49 POs.

PO, pulmonary ossification; C-setting, conventional setting with 512 × 512 matrix size and 0.5 mm slice thickness; UHR-setting, ultra-high-resolution setting with 2048 × 2048 matrix size and 0.25 mm slice thickness.

For Reader 1, at least one PO was seen with C-setting in 16 of 20 patients (80%) and with UHR-setting in 20 of 20 patients (100%). DPO was seen with C-setting in 3 of 20 patients (15%) and with UHR-setting in 16 of 20 patients (80%). DPO was seen more frequently with UHR-setting than with C-setting (*p* < 0.0001). With C-setting, scores were significantly higher in the right lower lobes than in the right middle lobes (*p* = 0.002). Scores were significantly higher in the left lower lobes than in the left upper (*p* = 0.001) and lingula lobes (*p* = 0.003). With UHR-setting, scores were significantly higher in the right lower lobes than in the right middle lobes (*p* = 0.01), and scores were significantly higher in the left lower lobes than left in the upper (*p* < 0.001) and lingula lobes (*p* = 0.008). For Reader 2, at least one PO was seen with each of C-setting and UHR-setting in 19 of 20 patients (95%). DPO was seen with C-setting in four of 20 patients (20%) and with UHR-setting in 6 of 20 patients (30%). There was no significant difference in the frequency of DPO between C-setting and UHR-setting (*p* = 0.48). With C-setting, scores were significantly higher in the right lower lobes than in the upper (*p* < 0.001) and middle lobes (*p* = 0.02). Scores were significantly higher in the left lower lobes than in the upper (*p* = 0.001) and lingula lobes (*p* < 0.001). With UHR-setting, scores were significantly higher in the right lower lobes than in the middle lobes (*p* = 0.01). Scores were significantly higher in the left lower lobes than in the upper (*p* = 0.02) and lingula lobes (*p* = 0.001).

Categories were significantly higher with UHR-setting than with C-setting (*p* < 0.001 for both readers). There was no significant difference between IPF and the other ILDs in terms of setting for either reader (*p* > 0.05).

**Figure 1:**
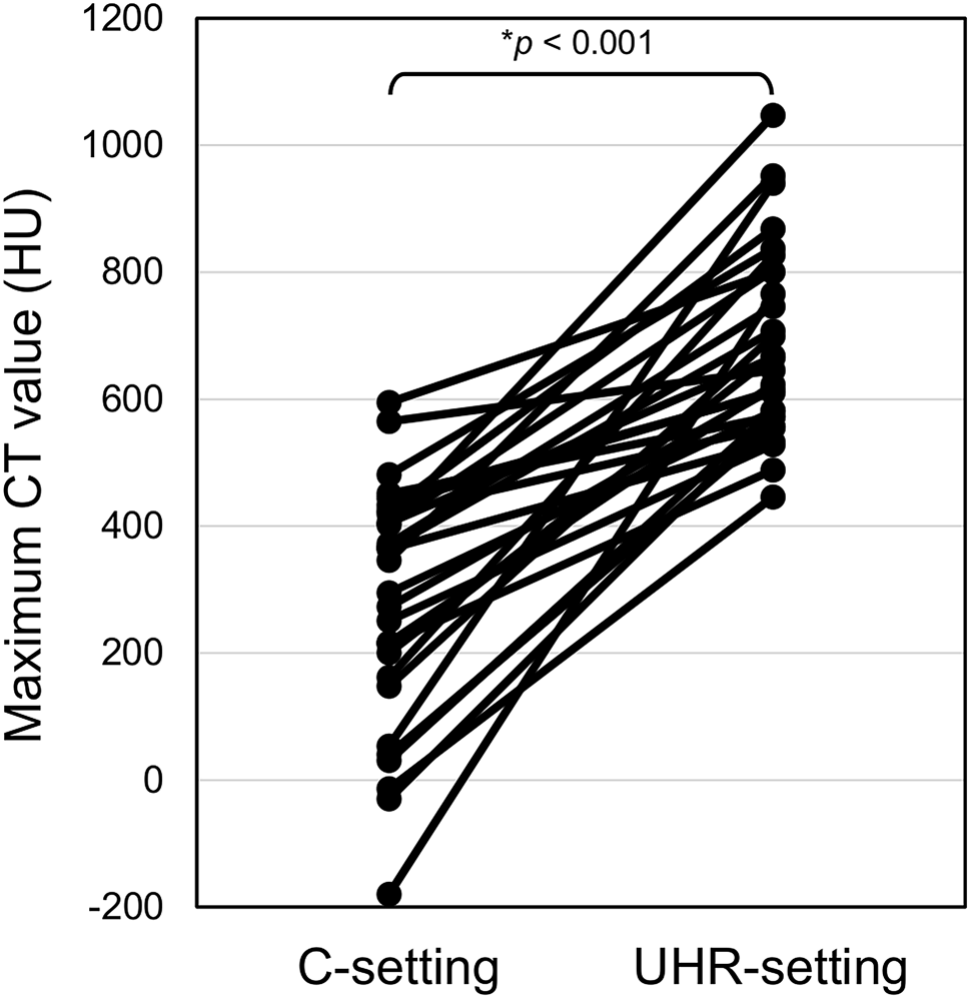
Analysis of maximum CT number. CT number was significantly higher with UHR-setting than with C-setting. *Wilcoxon signed rank test. C-setting, conventional setting with matrix of 512 × 512 and slice thickness of 0.5 mm; UHR-setting, ultra-high-resolution setting with matrix of 2048 × 2048 and slice thickness of 0.25 mm.

**Figure 2:**
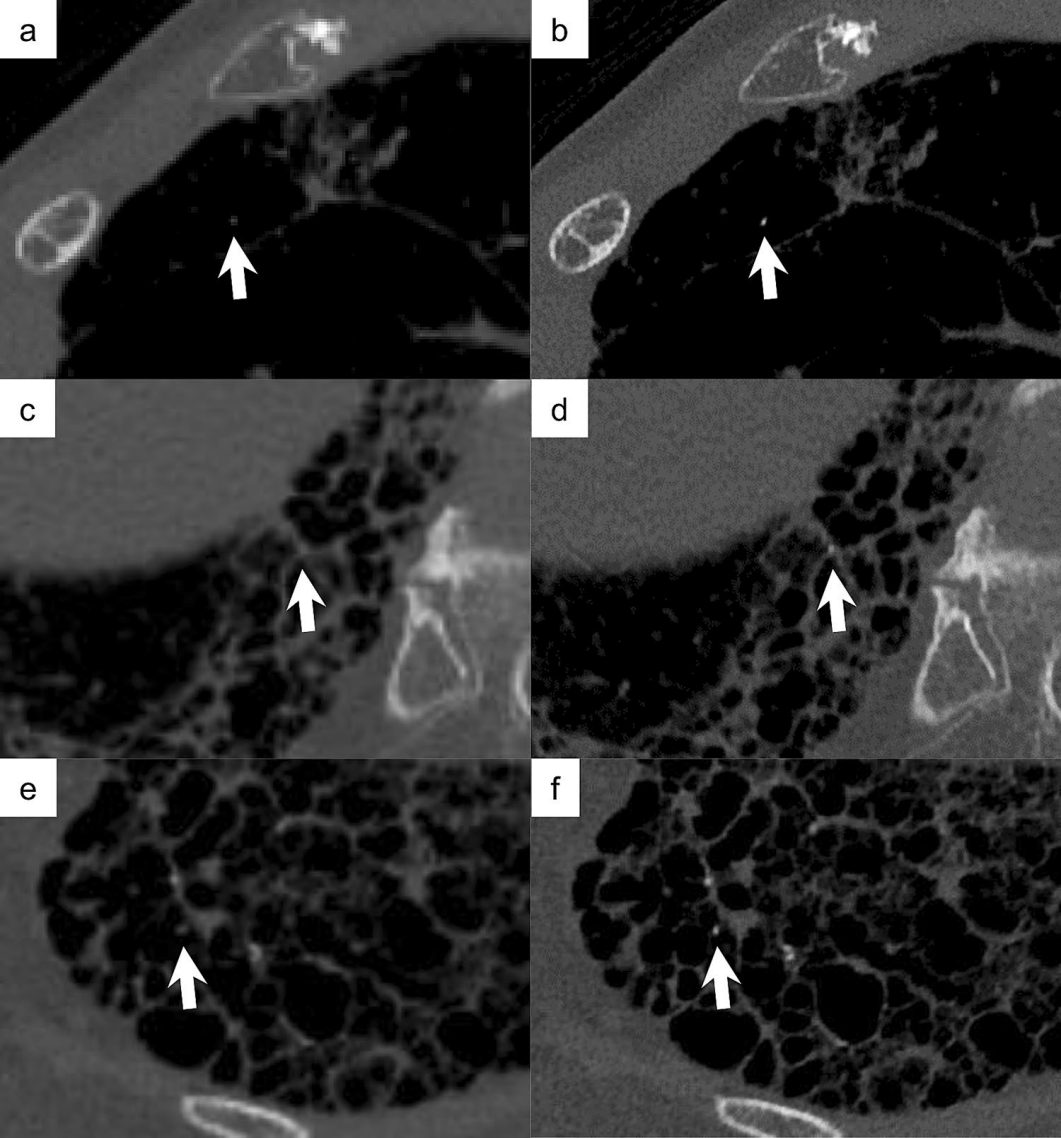
Representative cases of nodular PO on images obtained with C-setting (left side; matrix, 512 × 512; slice thickness, 0.5 mm) and with UHR-setting (right side; matrix, 2048 × 2048; slice thickness, 0.25 mm). (**a**, **b**) A 65-year-old woman with IPF; (**c**, **d**) a 77-year-old man with IPF; (**e**, **f**) a 61-year-old woman with systemic sclerosis. POs (arrows) are more clearly depicted in the images reconstructed with UHR-setting than in those with C-setting. The maximum CT number of the lesion indicated by each arrow was 53 HU in (**a**), 940 HU in (**b**), 140 HU in (**c**), 538 HU in (**d**), − 5 HU in (**e**), and 625 HU in (**f**). PO, pulmonary ossification; IPF, idiopathic pulmonary fibrosis; C-setting, conventional setting; UHR-setting, ultra-high-resolution setting.

For both readers, 33 lobes showed higher categories with UHR-setting than with C-setting, and no lobe showed a higher category with C-setting than with UHR-setting. When images with the two settings were compared side-by-side, the reader could not identify any PO lesion on the C-setting image that was detectable on the UHR-setting image in 4 of these 33 lobes. In the remaining 29 lobes, the reader measured the maximum CT number in one PO lesion in each lobe. The maximum CT number was 280 ± 191 HU with C-setting and 684 ± 150 HU with UHR-setting (Fig. 1). CT number was significantly higher with UHR-setting than with C-setting (*p* < 0.001). Representative cases are shown in Fig. 2.

## Discussion

In the present study, nodular POs were seen in ≥ 80% of patients who had honeycombing on UHRCT. DPO was seen in ≥ 15% of patients with C-setting and in ≥ 30% with UHR-setting. Scores tended to be higher in lower lobes than in the other lobes. PO category and maximum CT number were significantly higher with UHR-setting than with C-setting. These results indicate that nodular POs are more detectable when the UHR-setting is used. However, reconstruction setting had no effect on PO category between IPF and the other ILDs.

In our previous study of the effect of matrix size, a 2048 × 2048 image with a 320 mm FOV could depict a 0.15 mm slit, whereas a 512 × 512 image could not^9^. Theoretically, the lengths of a single pixel are 0.156 mm and 0.625 mm in 320 mm FOV images with matrix sizes of 2048 × 2048 and 512 × 512, respectively. Considering the differences between these spatial resolutions and theoretical pixel sizes, it is reasonable that UHR-setting images can detect finer structures and abnormalities than is possible on C-setting images. The present study compared UHR-setting and C-setting images obtained using the same UHRCT scanner; however, the differences in detection ability between images obtained using UHRCT and conventional CT remain unclear. The spatial resolution of UHRCT is reported to be 0.14 mm, whereas that of conventional CT is in the range 0.23–0.35 mm^9,17,18^. Moreover, 512 × 512 matrix size is more commonly used clinically, and larger matrix size is often unavailable on conventional CT. However, 512 × 512 matrix size with 320 mm FOV is not appropriate to demonstrate the maximum spatial resolution even on the conventional CT. Therefore, we consider that UHRCT with UHR-setting can detect more nodular POs compared with conventional CT.

In the present study, the prevalence of DPO was ≥ 30% on images reconstructed with UHR-setting, which is higher than that of 18.9% reported by Egashira et al. in IPF patients scanned with conventional high resolution CT and using the same definition of DPO^8^. Although there are differences between the studies in terms of patient background and sample size, the UHRCT scanner and UHR–setting used in our study might have enabled the higher detection rate.

The present results found no significant difference in PO category between IPF and other ILDs. The sample size in this study was too small for evaluation of the diagnostic ability for IPF. In the previous study by Egashira et al., PO scores were higher at biopsy in 31 patients with nodular ossification than in 88 patients without nodular ossification^8^. Further investigation using a larger sample size and including pathological examination is necessary to determine the diagnostic ability of UHRCT for IPF. In addition, we investigated patients with terminal fibrosis (honeycombing), but it is necessary to examine a group of patients with less advanced disease to see whether finding POs could be helpful in the differential diagnosis of early-stage ILD.

Interobserver agreement for the PO category was excellent with C-setting but moderate with UHR-setting. C-setting images are used clinically at our institution, but UHR-setting images are not. As the detection of PO is subjective, unfamiliarity with UHR-setting images may be the cause of this discrepancy.

In four lobes, the PO score was higher with UHR-setting than with C-setting for both readers, but in the subsequent evaluation, the reader could not identify POs on C-setting that were detectable on UHR-setting when the two setting images were compared side-by-side. This may be due to the subjective decision of PO detection that means the lesion can be overlooked on C-setting images, and which suggests that POs are often missed on C-setting images. In clinical practice, it is important that lesions are easy to detect because the reading time available to radiologists and clinicians is often limited. Images obtained with UHR-setting, on which POs are highly conspicuous, may therefore be of value in the clinical setting.

In the analysis of maximum CT number, the highest value with C-setting was larger than the lowest value with UHR-setting, even though the ROI was placed on a PO lesion which was not detectable on the image with C-setting but was detectable on that with UHR-setting. This may have occurred because the maximum CT number is generally influenced by noise and surrounding structures, and there can be a discrepancy between maximum CT number and the subjective impression.

This study had several limitations. The study design was retrospective and included patients at a single institution. As mentioned above, further investigations are needed to validate our study results and to evaluate the clinical usefulness of UHRCT. The detection of PO was subjective and we did not obtain pathological proof of the ossification. Furthermore, the diagnosis of ILD was not based on pathology. We did not evaluate dendriform pulmonary ossifications, and focused only on tiny lesions. We categorized the numbers of POs (classified as C0–C4) to analyze the extent PO lesions in the lobes, but this is not a widely used system. We used different iterative reconstruction (IR) settings for the UHR-setting and the C–setting: AIDR3D eStrong and eMild, respectively. eStrong generally has less noise and more blurring of the image compared with eMild. To compare the two types of reconstruction simply, we would ideally have used the same IR setting for both; however, due to the high level of noise in the 2048 × 2048 matrix image, we considered that eStrong would be more appropriate in the clinical setting. The difference in reconstruction algorithms might affect the quantification of CT value. However, it was assumed that eStrong setting resulted in a lower maximum CT value by blurring the image. Nevertheless, the UHR-setting images showed a higher maximum CT value than the C-setting in our results. In this study, we did not differentiate POs in the honeycombing and those in the normal lung. The advantage of UHRCT may be important in less advanced ILD rather than ILD with terminal fibrosis. There is the possibility that the surrounding area of POs might affect the visibility of POs.

In conclusion, nodular pulmonary ossifications were seen in 80% or more of patients with honeycombing on ultra-high-resolution CT and more easily detectable in images with UHR-setting than with C-setting, which may support clinical practice in the diagnosis of ILD.

## Abbreviations

AIDR3D: Adaptive iterative dose reduction 3D
CT: Computed tomography
C-setting: Conventional setting
FOV: Field of view
HU: Hounsfield unit
ILD: Interstitial lung disease
IPF: Idiopathic pulmonary fibrosis
PO: Pulmonary ossification
SD: Standard deviation
ROI: Region of interest
UHRCT: Ultra-high resolution CT
UHR-setting: Ultra-high resolution setting
UIP: Usual interstitial pneumonia
